# Supplementary material for: SET-M33 peptide as a selective in vitro antimicrobial agent against the porcine respiratory pathogen Glaesserella parasuis
Source: Microbiol Spectr. 2026 Feb 18;14(4):e03918-25. doi: 10.1128/spectrum.03918-25 (PMC13055275; doi:10.1128/spectrum.03918-25)
Supplement: Supplemental material — Table S1; Fig. S1 and S2. [file spectrum.03918-25-s0001.docx]

**Supplementary information for**

SET-M33 peptide as a selective *in vitro* antimicrobial agent against the porcine respiratory pathogen *Glaesserella parasuis*

[**Supplementary tables 2**](#_Toc209702145)

[**Supplementary table 1. List of strains used in study. 2**](#_Toc209702146)

[**Supplementary figures 3**](#_Toc209702147)

[**References 5**](#_Toc209702148)

# Supplementary tables

## Supplementary table 1. List of strains used in study.

| **Strain** | **Type** | **Source** |
| --- | --- | --- |
| *Streptococcus suis* P1/7 | Pathogenic | (1) |
| *Actinobacillus pleuropneumoniae* 4074 | Pathogenic | (2) |
| *Glaesserella parasuis* Nagasaki | Pathogenic | (3) |
| *Lactobacillus plantarum* KD9-5 | Commensal | (4) |
| *Streptococcus pluranimalium* LG3-6 | Commensal | (5) |
| *Rothia nasimurium* UK1-9 | Commensal | (5) |
| *Glaesserella parasuis* F9 | Commensal | (6) |
| *Neisseria shayeganii* GM3-3 | Commensal | (4) |
| *Staphylococcus aureus* EJ41-2 | Commensal | (4) |
| *Escherichia coli* NCM3722 | Sentinel strain | (7) |

# Supplementary figures


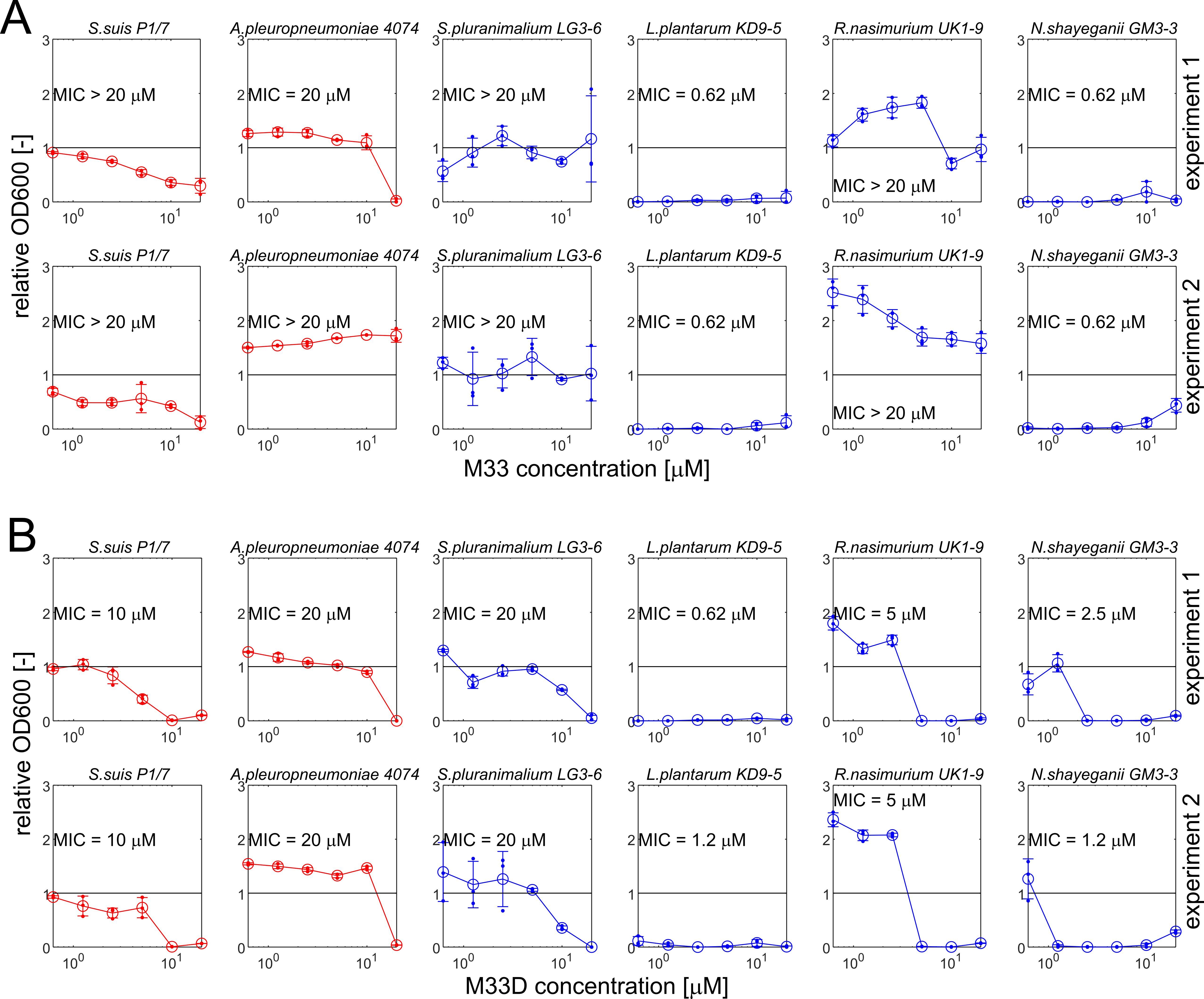


**Supplementary figure 1**. **Day-to-day reproducibility of MIC determination for SET-M33 (A) and SET-M33D (B).** Data shown are from two independent experiments performed on different days (top and bottom row in each panel). Relative OD600 (relative to untreated controls) after 24h incubation in BHI+ with different compound concentrations for each strain. Error bars denote standard deviation (n = 3), small circles denote individual replicate wells. In each case, the MIC was determined as the lowest compound concentration with a relative OD600 of <10%, and the absence of growth was confirmed by visual inspection of the culture plates. Red: selected reference pathogenic strains. Blue: selected commensal strains.


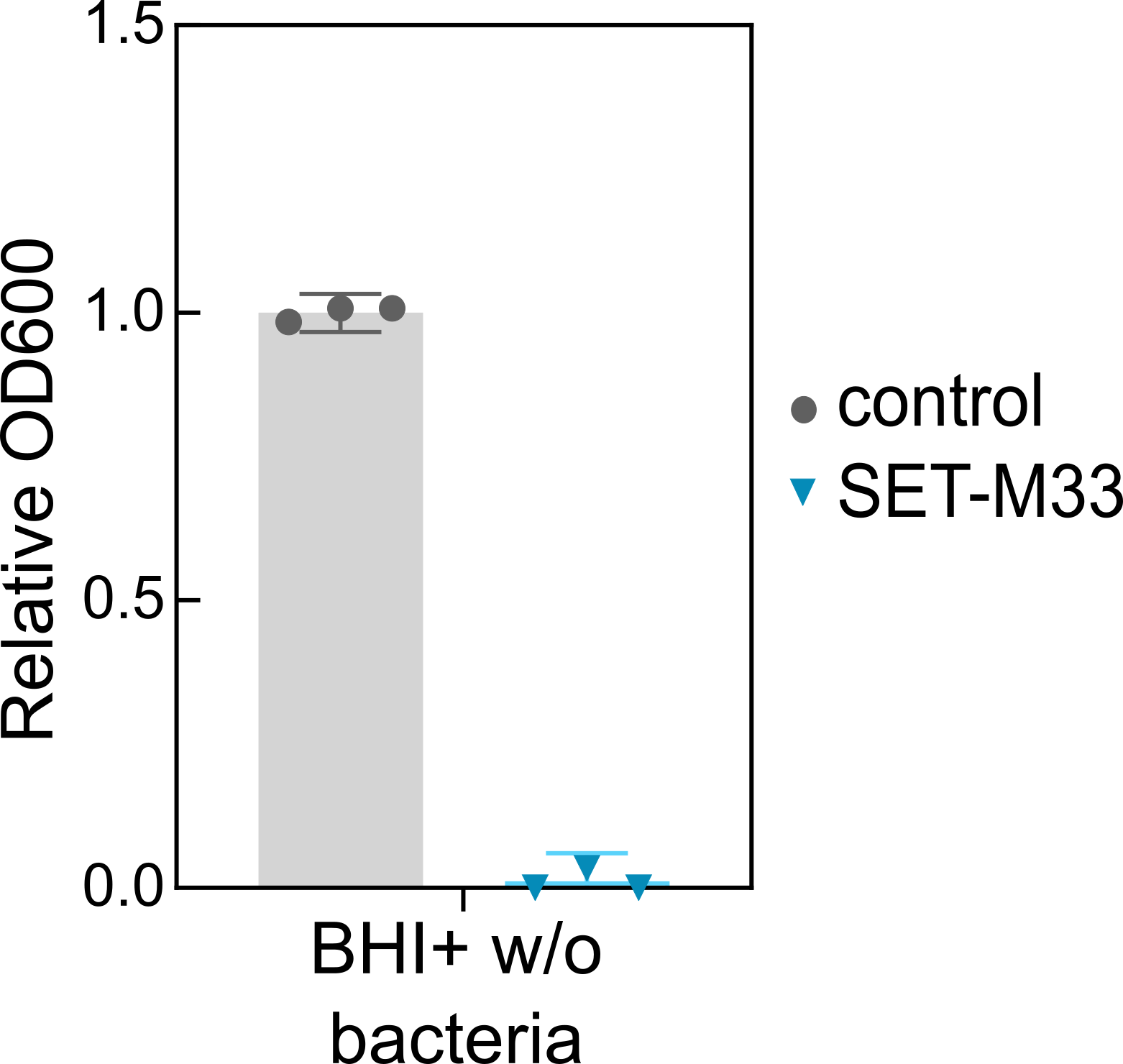


**Supplementary figure 2.** **SET-M33 does not get inactivated by prolonged (i.e. 24h) incubation in BHI+ in absence of bacteria.** Relative OD600 (relative to untreated controls) of SET-M33 susceptible sentinel strain (*E. coli* NCM3722) in cultivation media (BHI+) incubated either without (control) or with 10 μM SET-M33 at 37ºC for 24h. Error bars denote standard deviation (n = 3), small circles denote individual replicate wells.

# References

1. Fittipaldi N, Xu J, Lacouture S, Tharavichitkul P, Osaki M, Sekizaki T, et al. Lineage and Virulence of *Streptococcus suis* Serotype 2 Isolates from North America. Emerg Infect Dis. 2011 Dec;17(12):2239–44.

2. Frey J, Nicolet J. Purification and partial characterization of a hemolysin produced by *Actinobacillus pleuropneumoniae* type strain 4074. FEMS Microbiology Letters. 1988 Sept;55(1):41–5.

3. Amano H, Shibata M, Kajio N, Morozumi T. Pathologic Observations of Pigs Intranasally Inoculated with Serovar 1, 4 and 5 of *Haemophilus parasuis* Using Immunoperoxidase Method. J Vet Med Sci. 1994;56(4):639–44.

4. Bonillo-Lopez L, Rouam-el Khatab O, Obregon-Gutierrez P, Florez-Sarasa I, Correa-Fiz F, Sibila M, et al. *In vitro* metabolic interaction network of a rationally designed nasal microbiota community. iScience. 2025 July 14;28(8):113114.

5. Blanco-Fuertes M, Sibila M, Franzo G, Obregon-Gutierrez P, Illas F, Correa-Fiz F, et al. Ceftiofur treatment of sows results in long-term alterations in the nasal microbiota of the offspring that can be ameliorated by inoculation of nasal colonizers. anim microbiome. 2023 Oct 20;5(1):53.

6. Olvera A, Cerdà-Cuéllar M, Aragon V. Study of the population structure of *Haemophilus parasuis* by multilocus sequence typing. Microbiology. 2006 Dec 1;152(12):3683–90.

7. Soupene E, Van Heeswijk WC, Plumbridge J, Stewart V, Bertenthal D, Lee H, et al. Physiological Studies of *Escherichia coli* Strain MG1655: Growth Defects and Apparent Cross-Regulation of Gene Expression. J Bacteriol. 2003 Sept 15;185(18):5611–26.
